# Assessment of the anesthetic effect of modified pentothal sodium solution on Sprague-Dawley rats

**DOI:** 10.1515/biol-2022-0050

**Published:** 2022-05-12

**Authors:** Xianzhen Chen, Shiqing Wang, Youjiong Li, Chunjin Lin, Xiaofang Liu

**Affiliations:** Department of Anesthesia, Women and Children’s Hospital Affiliated to Xiamen University, 10 Zhenhai Road, Siming, Xiamen, 361001, People’s Republic of China

**Keywords:** pentothal sodium, ketamine, anesthetic effect, intraperitoneal injection

## Abstract

Clinically, pentothal sodium has been widely used for primary and general anesthesia induction. Also, it has been used to effectively inhibit convulsion. Pentothal sodium has a strong inhibitory effect on the respiratory center, excessive drug administration, and rapid dose rate that cause death of experimental animals on the respiratory depression. This study used a modified pentothal sodium solution to investigate its anesthetic effect. The pentothal sodium solution was modified based on pentothal sodium upon additions of magnesium sulfate, propylene glycol, and pure ethanol. The anesthetic effect of the modified pentothal sodium on Sprague–Dawley (SD) rats was investigated by comparing traditional pentothal sodium and ketamine; 60 SD rats were randomly divided into three groups. Each group was treated with traditional pentothal sodium, modified pentothal sodium, or ketamine, respectively, via intraperitoneal injection. The symptoms of experimental rats were observed, and onset time and anesthetic time were both recorded. The data were analyzed using statistical software. There were no significant differences in onset time and anesthetic time between the three groups. The variation of onset time and anesthetic time of the group treated with modified pentothal sodium was shorter than that of the other two groups. Furthermore, the number of anesthetic rats after the first injection was significantly higher than that of the other two groups. The modified pentothal sodium is capable of providing a stable anesthetic effect. The function and effect are much better than traditional pentothal sodium and ketamine.

## 1 Introduction

Animal experiments have been widely used in the studies of disease modeling, pharmaceutics, praxeology, and nutriology. Anesthesia is a very important section of animal experiments. The effects of anesthesia directly affect the progress of animal surgery and the accuracy of the experimental observation and data [1,2,3,4,5,6,7]. The requirements for anesthesia experiments are as follows: fast operation, long duration of anesthesia, and the experimental animals keep stable and quiet during anesthesia [8,9]. An unfavorable anesthetic effect will lead to the animal screaming and making noise, which will affect the accuracy of the operation and cause accidental injury to the experimental animals.

Pentothal sodium is a barbituric acid derivative belonging to the central sedative-hypnotics [1,2,3,4,5,6,7,8,9,10]. Pentothal sodium has many advantages, such as rapid anesthetic operation and stable anesthetic effect [11,12]. Currently, the pentothal sodium solution is widely used in experimental research and teaching because it meets the general requirements for anesthesia of experimental animals. However, pentothal sodium has a strong inhibitory effect on the respiratory center. Excessive drug administration and rapid dose rate will cause the death of experimental animals due to respiratory depression [13,14]. Previous studies about the anesthetic effect of pentothal sodium focused on the dose of the solution, anesthesia time, and comparison of other anesthetics [15,16,17]. However, the main problems such as unstable dosage, large fluctuations of anesthesia time, and excessive administration were less discussed.

In this study, the modified pentothal sodium anesthetic was prepared based on pentothal sodium and upon additions of magnesium sulfate, propylene glycol, and pure ethanol. These additives will enhance the solubility of pentothal sodium and promote the percutaneous absorption of drugs [15,16,17]. The results of comparing the anesthetic effect with traditional pentothal sodium and ketamine indicated that the modified pentothal sodium is better than the other two anesthetics. Based on the current study results, the modified pentothal sodium decreased the probability of death of animals. Therefore, it can be widely applied in clinical and experimental research.

## 2 Materials and methods

### 2.1 Chemicals and anesthetics

Pentothal sodium and ketamine were purchased from Sinopharm Chemical Reagent (Shanghai) Co. Ltd. Magnesium sulfate and pure ethanol were purchased from Sigma-Aldrich. The modified pentothal sodium solution was prepared by dissolving pentothal sodium into a solvent of mixed physiological saline, propylene glycol, and ethanol.

### 2.2 Animal experiments

About 60 SPF-grade Sprague–Dawley (SD) rats (age, 5–6 weeks; weight, 180–200 g) were purchased from the Animal Center of Beijing Medical University. The sexual ratio of male and female rats is 1:1. The rats were randomly divided into three experimental groups for anesthetic experiments using modified pentothal sodium (Group I), traditional pentothal sodium (Group II), and ketamine (Group III), with 20 rats in each group. Temperature and humidity were 25 ± 1°C and 50–60%, respectively. Illumination time and dark time were both 12 h. There were no restrictions on drinking water and feeding.


**Ethical approval:** The research related to animal use has complied with all the relevant national regulations and institutional policies for the care and use of animals.

## 3 Experimental method

Each rat was dosed via intraperitoneal injection at 45 mg/kg body weight for Group I, 50 mg/kg body weight for Group II, and 250 mg/kg body weight for Group III, respectively [18]. The status of rats was observed after dosing. Onset time was defined as the period from the anesthetic intraperitoneally injected into a rat to the beginning of anesthetic symptoms. Anesthesia time was defined as the period from the beginning of anesthetic symptoms to the recovery of the rats.

### 3.1 Statistical analysis

All data were expressed using *χ* ± *s*, the differences between the groups were compared using single-factor analysis of variance, SPSS 11 software was used for variance analysis of the data, and Bonferroni correction between the groups.

## 4 Results

**Figure 1:**
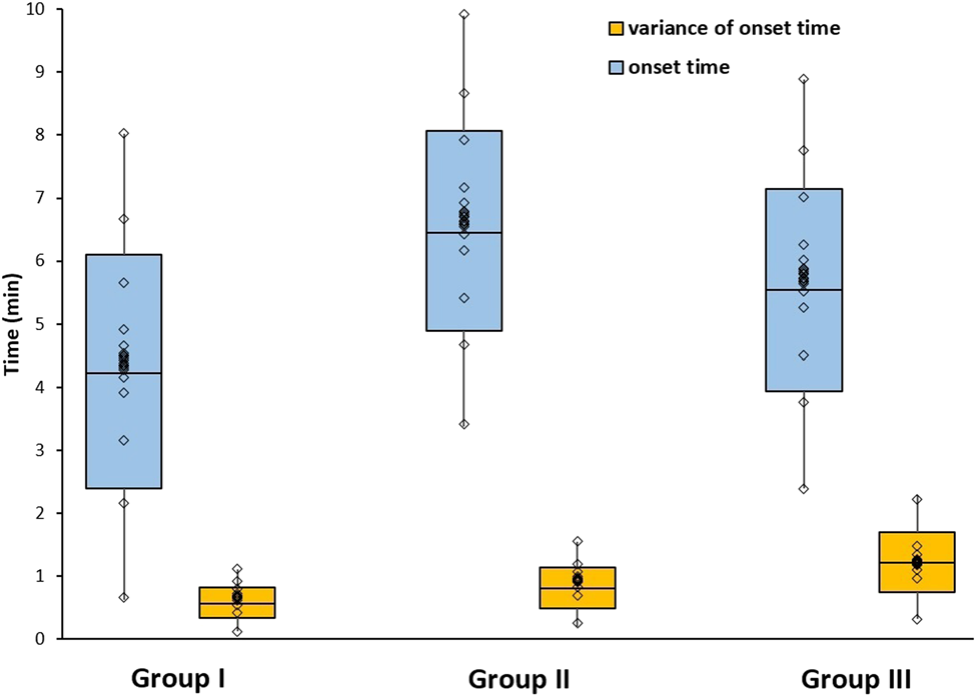
Comparison between onset time and variance of onset time for three anesthetics.

The onset times of different anesthetics are shown in Figure 1. The statistical analysis showed no significant difference between Groups I and II and Groups I and III, respectively (*P* > 0.05). The variance of onset time for Group I (modified pentothal sodium method) is the smallest among the three groups. The statistical analysis showed a significant difference between Groups II and III data compared with Group I, suggesting that the onset time of modified pentothal sodium is more stable than in the other two groups (*P* < 0.01).

**Figure 2:**
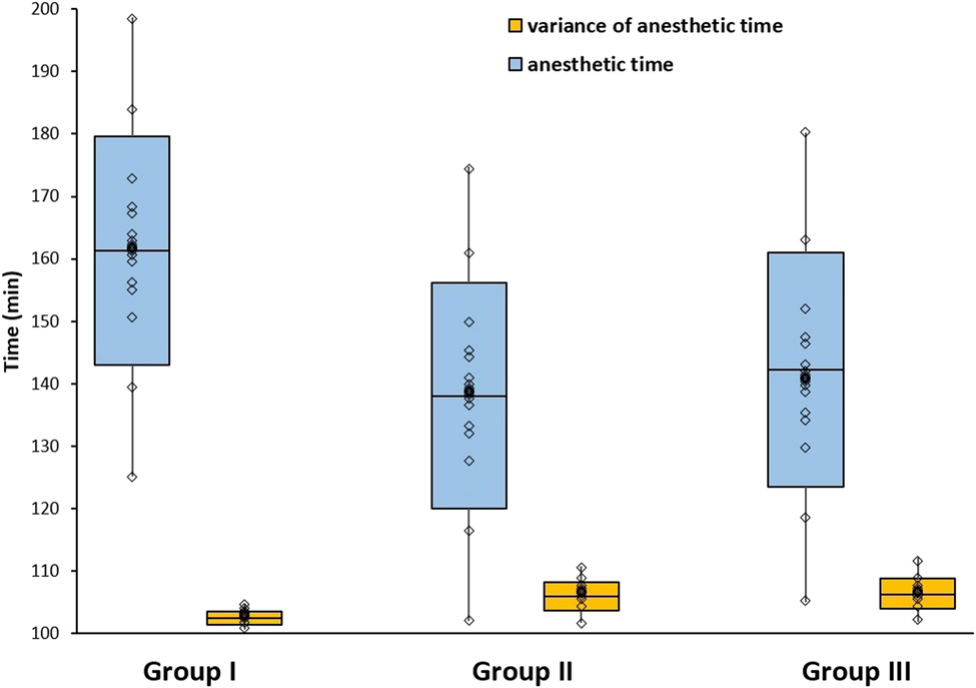
Comparison between anesthetic time and variance of anesthetic time for three anesthetics.

The anesthetic times of different anesthetics are shown in Figure 2. Statistical analysis showed no significant difference between Groups I and II and Groups I and III, respectively (*P* > 0.05). Therefore, the anesthetic times of the three experimental groups were similar. It was found that the variance of anesthetic time in Group I is the smallest. Statistical analysis showed significant differences between Groups I and II and Groups I and III, respectively, suggesting that the anesthetic time of modified pentothal sodium is shorter than the other two groups with a good consistency (*P* < 0.01).

The stability and safety of three anesthetic methods were evaluated by comparing the abnormal behaviors induced by different anesthetics. The number of successful cases, cases that need additional anesthesia, and deaths in each group were statistically analyzed. The data are listed in Table 1. No deaths were observed in Group I, and only two cases needed additional anesthesia. However, the death cases and cases that needed additional anesthesia in Groups II and III are seven cases and six cases, respectively. The statistical analysis showed a significant difference between Groups I and II (*χ* ^2^ = 3.752, *P* < 0.05). No significant difference was found between Groups II and III (*χ* ^2^ = 3.218, *P* > 0.05).

**Table 1:** Comparison of the abnormal behaviors induced by different anesthetics for SD rats

Group	Number of successful cases by first anesthesia	Number of cases need additional anesthesia	Number of death cases	*χ* ^2^
I	18	2	0	—
II	13	5	2	3.752
III	14	5	1	3.218

## 5 Discussion

The traditional pentothal sodium has several disadvantages in anesthesia application. It is slightly soluble in water; the aqueous solution is not stable; and it is easy to crystallize, resulting in an unstable effective concentration. The traditional pentothal sodium cannot be preserved for a long time [4,5,6,7]. In order to overcome these disadvantages, propylene glycol was added to water to prepare a complex solvent to enhance the solubility and avoid precipitation of pentothal sodium, and the solution will be more stable. Pure ethanol was also added to the solvent. Like propylene glycol, ethanol is a commonly used transdermal enhancer, which increases the solubility of drugs and promotes the transdermal absorption of drugs. In addition, ethanol at high concentration can inhibit the advanced function of the brain, leading to an indirect enhanced anesthetic effect by pentothal sodium. This study reduced the effective dosage and concentration of pentothal sodium in modified pentothal sodium anesthetic [4]. Previous studies have demonstrated that excess pentothal sodium anesthetic caused the death of experimental rats due to respiratory inhibition via gamma-aminobutyric acid receptors [11]. The decrease in effective dose will reduce the side effects and improve the safety of experimental animals. This study also showed that the dosage of 45 mg/kg body weight for modified pentothal sodium could reach the same or even better effect as traditional pentothal sodium at 50 mg/kg body weight. There are no significant differences between the onset time and the anesthetic time of the two pentothal sodium drugs. Most importantly, the concentration of modified pentothal sodium is very consistent, and the anesthetic effect is stable with very small fluctuations. The number of successful cases by first anesthesia using modified pentothal sodium is higher than the other two anesthetics, and this new anesthetic was found to reduce invalid anesthesia and death cases. Moreover, the variance of onset time and anesthesia time is less than the other two groups. It is well known that the instability of anesthesia time will affect the physiological function of experimental animals and the final results of experiments. The uniform anesthesia time caused by modified pentothal sodium will significantly minimize the side effects of anesthesia, further ensuring the stability of experimental results.

To sum up, in order to overcome some disadvantages of traditional pentothal sodium, three methods were used: first, the addition of propylene glycol, pentothal sodium is slightly soluble in water, resulting in an unstable aqueous solution; pentothal sodium is easy to precipitate and crystallize. Therefore, the effective concentration of the anesthesia is unstable, and the solution cannot be stored for an extended period. As an excellent semi-polar pharmaceutical auxiliary solvent, propylene glycol was mixed with water to prepare a complex solvent, which significantly enhanced the solubility of pentothal sodium, avoiding the precipitation of pentothal sodium. Furthermore, propylene glycol has few side effects, with the capacity of improved viscosity and moisture absorption. It will significantly increase the transdermal absorption of pentothal sodium to a certain extent and improve the drug utilization, as previously suggested by Fragoza and Alston [18]. Second, with the addition of ethanol, the function is similar to that of propylene glycol. As a commonly used transdermal absorption enhancer, ethanol can not only increase the solubility of drugs but also promote the percutaneous absorption of drugs. In addition, a certain concentration of ethanol can inhibit the advanced function of the brain and indirectly enhance the anesthetic effect of pentothal sodium. Last but not least, a decreased concentration of pentothal sodium was applied to reduce its side effects and to improve its safety [19].

There are several limitations of this study, such as the limited number of animals used and the operators who conducted the anesthesia. These need to be further studied in future work.

## 6 Conclusion

Modified pentothal sodium solution was used as a complex anesthetic containing pentothal sodium, magnesium sulfate, propylene glycol, and pure ethanol. The new drug overcame the common disadvantages of traditional pentothal sodium and reduced the effective dose of pentothal sodium. The accuracy of experimental results is improved using the modified pentothal sodium solution. This new anesthetic has some advantages, with improved safety and strength of anesthesia. The onset time and anesthesia time are both stable with slight fluctuation.
